# Pressure-Induced Phase Transitions and Electronic Structure Evolution of Ba_4_Au

**DOI:** 10.3390/ma18163728

**Published:** 2025-08-08

**Authors:** Xinyu Wang, Qun Wei, Jing Luo, Xiaofei Jia, Meiguang Zhang, Xuanmin Zhu, Bing Wei

**Affiliations:** 1 School of Physics, Xidian University, Xi’an 710071, China; 2 College of Physics and Optoelectronic Technology, Baoji University of Arts and Sciences, Baoji 721016, China; 3 School of Information, Guizhou University of Finance and Economics, Guiyang 550025, China

**Keywords:** crystal structure prediction, gold-based alloys, phase transitions, first-principles calculations

## Abstract

Considering previous studies on the high-pressure phases and compressibility of Ba–Au alloys with stoichiometries Au_2_Ba, AuBa, and Au_2_Ba_3_, the concentration of the alkaline-earth metal Ba increased, and a particle-swarm optimization algorithm was employed to conduct comprehensive structure searches for the Ba_4_Au compound at 0, 10, 20, and 50 GPa. First-principles calculations were subsequently carried out to investigate its structural evolution and electronic properties under compression. Enthalpy-difference calculations indicate that the *I*4*/mmm* phase of Ba_4_Au transforms to the *Cmmm* phase at approximately 0.4 GPa. As pressure increases above 5.7 GPa, the *I*4*/m* structure becomes energetically more favorable than *Cmmm*-Ba_4_Au, indicating that the *Cmmm* phase transforms to the *I*4*/m* phase at 5.7 GPa. Both phase transitions are first-order and accompanied by discernible volume collapses. Additionally, a comparative analysis of the electronic properties of Ba_4_Au was performed before and after the phase transitions. In this study, theoretical guidance is provided for the exploration of the high-pressure structural evolution of Ba_4_Au, and critical insights are offered regarding the changes that occur in its physical and chemical properties under compression.

## 1. Introduction

Gold-based alloy systems have garnered remarkable attention owing to their enhanced performance, cost-effectiveness, and broad application prospects compared to pure gold. Previous studies have demonstrated that gold-based alloys have extensive applications in catalysis, electronic transport, and optics [1,2,3,4]. For example, Zhao et al. [5] reported a phase-stabilized synthesis method for preparing Au_3_Cu alloy nanocrystals. The resulting Au–Cu alloy exhibited considerably enhanced catalytic activity in CO_2_ electroreduction reactions. Ishikawa et al. [6] investigated the properties of Au–In alloys, and Baranov et al. [7] examined the superconductivity of Au–Pb alloys. Both studies found that the superconducting behavior of Au–In and Au–Pb alloys is markedly enhanced compared to that of pure gold [6,7,8]. Lu et al. [9] explored the properties of Au–Al alloys and found that the AuAl_2_ alloy exhibits a distinctive purple appearance owing to the synergistic effect of plasmons and interband transitions, with implications for its application in jewelry coloration and optical filtering. Refs. [10,11,12] pointed out that some alkali metal aurides, such as KAu, RbAu, and CsAu, exhibit semiconducting behavior despite being composed entirely of metallic elements. Therefore, it is worthwhile to investigate novel gold-based alloy materials that may possess unique properties.

Furthermore, it has been recognized that pressure is an important means of tuning the microstructure of materials. It can induce chemical bond contractions and atomic arrangement reconstruction, thereby altering physical properties or triggering phase transitions in crystal structures [13,14,15,16,17]. Notably, at ambient pressure, the chemical behavior of Ba is governed by its 6*s*^2^ valence electrons, resulting in a typical +2 oxidation state and ionic bonding characteristic. However, under high-pressure conditions, its electronic structure undergoes a marked change. When pressure exceeds several tens of GPa, the energy levels of Ba’s inner 5*p* and 5*d* orbitals considerably increase owing to compression, enhancing *s–p* orbital hybridization and potentially driving the oxidation state beyond conventional limits [18,19,20,21,22]. For example, in high-pressure BaF_n_ (*n* = 3, 4, and 5) compounds, Ba exhibits oxidation states from +3 to +5 by opening its inert 5*p* shell, with an electronic configuration approaching that of *p*-block elements [21]. In high-pressure Ba_3_Ch_2_ (Ch = S, Se, and Te) compounds, Ba displays mixed +1 and +2 valence states and shows *d*-block-like magnetic behavior [22]. Li et al. investigated the high-pressure behavior and electronic properties of BaAu compounds, demonstrating that the ambient-pressure *Pnma*-BaAu structure transformed into a cubic structure with *Fd-*3*m* symmetry at approximately 12 GPa [23]. This pressure-induced orbital reconstruction imparts Ba with pseudo-transition metal characteristics, enabling unconventional stoichiometries and bonding motifs in alloy systems. Owing to its unique high-pressure response and strong relativistic effects, the alkaline-earth metal Ba serves as an ideal model for exploring novel alloy compounds under extreme conditions. Therefore, it is meaningful to investigate the high-pressure phases of the Ba–Au alloy system.

In recent years, various algorithms have been developed for predicting material structures, such as minima hopping [24], metadynamics [25], the genetic algorithm [26], particle swarm optimization [27], and the random sampling method [28]. Among these, the CALYPSO structure prediction method, based on the particle swarm optimization algorithm, has been demonstrated as an effective approach for discovering new stable compounds based on known chemical compositions and external conditions. Its success has been validated in predicting the stable structures of a wide range of systems [29,30,31]. Recently, Li et al. [32] performed structure searches on the Au*_x_*Ba*_y_* systems (*x* = 1, 2; *y* = 1; *x* = 2, *y* = 3) at 0, 50, and 100 GPa using the CALYPSO structural prediction method, investigating their thermodynamic stability and compressibility under varying pressures, though they did not address the case of y = 4. Inspired by their work, we increased the Ba content and employed the CALYPSO crystal structure prediction method to identify stable Ba_4_Au structures at 0, 10, 20, and 50 GPa. Ba_4_Au is a new Ba-rich compound that does not appear in the Ba−Au system. Compared to combinations with lower Ba content, such as BaAu and Au_2_Ba, Ba_4_Au has a much higher Ba content and, consequently, a greater number of Ba–Au bonds. In high-pressure studies of BaAu, significant overlap between Ba 5*d* and Au 6*p* orbitals drives charge transfer and orbital hybridization [23]. In Ba_4_Au, this orbital mixing is expected to be even more extensive and pronounced, potentially leading to more complex electronic-structure evolution and even inducing a metal–semiconductor phase transition. This study focuses on using theoretical calculations and structure prediction to explore the pressure-induced phase transition pathways of Ba_4_Au in the 0–50 GPa range, revealing the evolution of its crystal structure and electronic properties. This study not only fills the research gap on Ba-rich compounds in the Ba–Au alloy system but also suggests that the Ba_4_Au structure has the potential to exhibit more prominent properties than structures with lower Ba contents in terms of electronic state control and pressure-induced transition behavior. It is worth noting that this study is purely theoretical and predictive in nature, with no experimental comparisons available at present. However, the results show a clear trend and can provide guidance for future experimental design and verification.

## 2. Computational Details

To identify the most stable crystal structure of Ba_4_Au, we employed the CALYPSO crystal structure prediction method, which is based on a particle swarm optimization (PSO) algorithm for global structure searching [33,34,35]. This study conducted variable-cell structure searches for Ba_4_Au at 0, 10, 20, and 50 GPa. The population size was set to 50 per generation, and the first generation was generated randomly under symmetry constraints. In each generation, 60% of the structures were obtained by the PSO operation of crystal structures with lower enthalpies in the previous generation, while the remaining 40% were produced randomly to ensure structural diversity. Thirty generations were sampled to ensure the convergence of the search. After the CALYPSO search, low-energy candidate phases were selected and further refined using the Vienna Ab initio Simulation Package (VASP5.4.4) [36] for structural optimization and related property calculations. Electron–ion interactions were described using the projector augmented wave method [37], and exchange–correlation effects were treated with the Perdew–Burke–Ernzerhof functional within the generalized gradient approximation [38,39]. A plane-wave cutoff energy of 600 eV and a Monkhorst–Pack *k*-point grid spacing of 2π × 0.02 Å^−1^ were employed to ensure total energy convergence within 1 × 10^−5^ eV/atom. Furthermore, the electron localization function (ELF) [40,41] was calculated using VASP. To assess dynamic stability, phonon dispersion calculations were performed using density functional perturbation theory as implemented in the PHONOPY code [42,43,44,45], with a force convergence threshold of 1 × 10^−7^ eV/Å.

## 3. Results and Discussion

To identify potentially stable Au*_x_*Ba*_y_* compounds, we extended previous work by increasing the Ba concentration. We performed comprehensive structure searches on the Ba_4_Au compound (corresponding to *x* = 1, *y* = 4) at 0, 10, 20, and 50 GPa. The ground state phase at 0 GPa was *I*4/*mmm*-Ba_4_Au, and that at 10, 20, and 50 GPa was *I*4/*m*-Ba_4_Au. The thermodynamic stability of each Au–Ba compound relative to elemental Au and Ba at a set pressure was quantified by the formation enthalpy [32] (∆*H*), defined as follows:

(1) $$\Delta H=\frac{H(Au_{x}Ba_{y})-xH(Au)-yH(Ba)}{x+y}$$

Here, *H*(Au*_x_*Ba*_y_*) denotes the total enthalpy of the compound while *H*(Au) and *H*(Ba) represent the enthalpies of single Au and Ba atoms in crystals; *x* and *y* denote the numbers of Au and Ba atoms in the unit cell, respectively.

**Figure 1:**
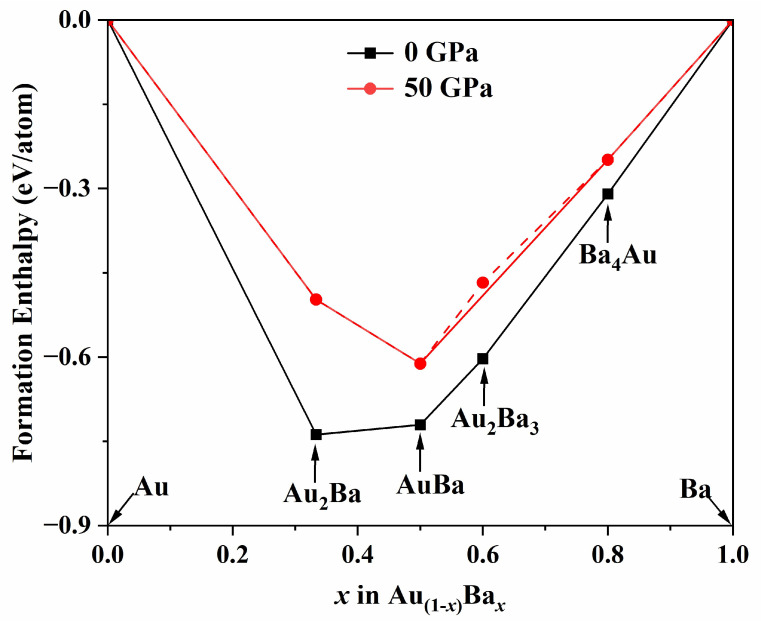
Phase stabilities of various Au*_x_*Ba*_y_* compounds at 0 and 50 GPa. Compounds corresponding to data points located on the convex hull are thermodynamically stable, while those on the dashed lines represent that the alloys are unstable with respect to their decomposition into elements or other stoichiometries.

Combining the structural data reported in the literature [32], we further refined the convex-hull diagram for Au*_x_*Ba*_y_* compounds, as shown in Figure 1. Table S2 presents the formation enthalpies of Au*_x_*Ba*_y_* (*x* = 1, *y* = 1, 4; *x* = 2, *y* = 1, 3) compounds at various pressures. Based on the convex-hull data, structures that exist on the solid line are thermodynamically stable at the corresponding pressure. However, points inside the convex polygon are unstable and can decompose into other compounds or elemental forms. Notably, the Ba_4_Au structure lies on the solid line at 0 and 50 GPa, indicating its thermodynamic stability under these conditions. This suggests a previously unreported stable Ba_4_Au phase. The phonon spectra of other predicted stable Ba_4_Au phases are shown in Figure S1.

**Figure 2:**
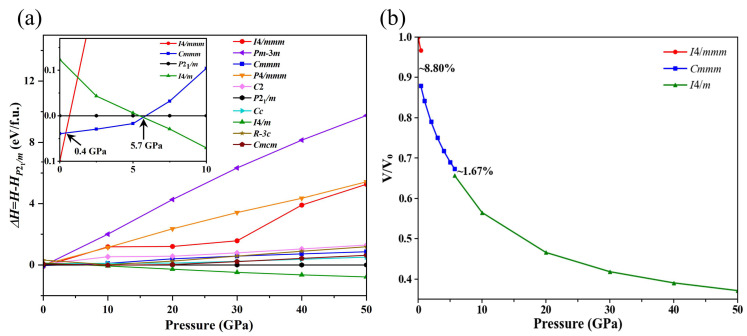
(**a**) Enthalpy (relative to *P*2_1_*/m*) as a function of external pressure for selected structures of Ba_4_Au in different symmetries. The inset shows an enlarged view of enthalpy differences for the four lowest-enthalpy phases in the 0–10 GPa region. (**b**) Equations of state for Ba_4_Au in the *I*4*/mmm*, *Cmmm*, and *I*4*/m* phases.

**Figure 3:**
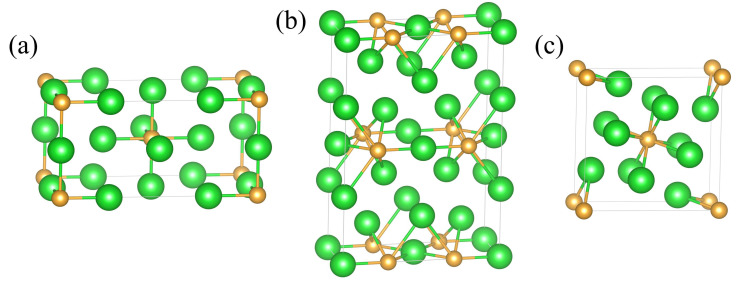
Crystal structures of the (**a**) *I*4*/mmm* (0 GPa), (**b**) *Cmmm* (5 GPa), and (**c**) *I*4*/m* (10 GPa) phases of Ba_4_Au (Ba in green atoms and Au in gold atoms).

To investigate the potential crystal structure transitions of Ba_4_Au in the 0~50 GPa pressure range, we used a swarm-intelligence-based CALYPSO structure search method in conjunction with the VASP code to identify the structures of Ba_4_Au at pressures of 0, 10, 20, and 50 GPa, respectively. In this study, we selected the 50 lowest enthalpy Ba_4_Au crystal structures predicted by the CALYPSO (version 6.0) package at each pressure, performed higher-precision structural optimizations, and calculated their elastic constants and phonon spectra, obtaining ten stable structures with the lowest energies (*I*4*/mmm*, *Pm-*3*m*, *Cmmm*, *P*4*/mmm*, *C*2, *P*2_1_*/m*, *Cc*, *I*4*/m*, *R-*3*c*, and *Cmcm*). Then, we plotted the enthalpy variations in these structures relative to the *P*2_1_*/m*-Ba_4_Au phase as a function of pressure (Figure 2a). This analysis revealed pressure-induced phase transitions among the candidate polymorphs. At ambient pressure, *I*4*/mmm*-Ba_4_Au has the lowest energy. Above 0.4 GPa, the *Cmmm* phase becomes energetically favorable, indicating that the *I*4*/mmm* phase of Ba_4_Au transforms to the *Cmmm* phase at approximately 0.4 GPa. As pressure increases, above 5.7 GPa, the *I*4*/m* structure becomes energetically more favorable than *Cmmm*-Ba_4_Au, indicating that the *Cmmm* phase transforms to the *I*4*/m* phase at 5.7 GPa. Figure 2b presents the volume–pressure curves obtained by fitting a third-order Birch–Murnaghan equation of state. Both the *I*4*/mmm*⟶*Cmmm* and *Cmmm*⟶*I*4*/m* transitions exhibited first-order characteristics, as indicated by significant volume collapses of 8.80% and 1.67%, respectively. The *I*4*/mmm* and *I*4*/m* phases adopted tetragonal symmetry. In these phases, Au atoms occupy the eight corners and body-center positions of the cell, with Ba atoms arranged around them (Figure 3a,c). In contrast, the *Cmmm* phase belongs to the orthorhombic system and exhibits distinct layering: each layer of Au atoms is surrounded by several Ba atoms (Figure 3b).

**Figure 4:**
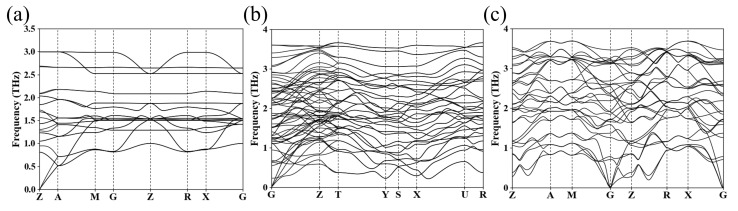
Phonon spectra of (**a**) *I*4*/mmm* (0 GPa), (**b**) *Cmmm* (5 GPa), and (**c**) *I*4*/m* (10 GPa) phases of Ba_4_Au.

Table 1 lists detailed structural parameters for the *I*4*/mmm*, *Cmmm*, and *I*4*/m* phases of Ba_4_Au for further study. Table S1 in the Supplementary Materials provides detailed structural information for other Ba_4_Au compounds. Figure 4 presents the phonon dispersion curves for the *I*4*/mmm*, *Cmmm*, and *I*4*/m* phases of Ba_4_Au. In each case, all phonon eigenfrequencies across the Brillouin zone were positive, confirming the dynamic stability of these structures.

**Figure 5:**
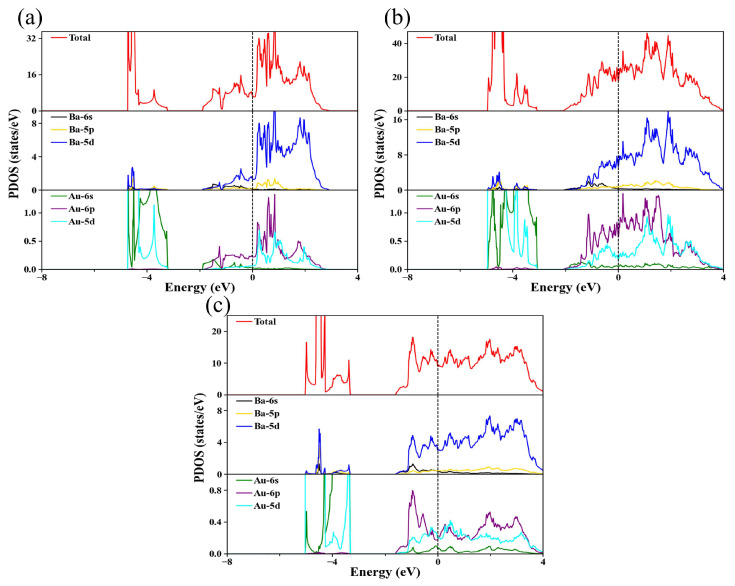
Total and partial density of states of Ba_4_Au: (**a**) *I*4*/mmm* phase at 0 GPa, (**b**) *Cmmm* phase at 5 GPa, and (**c**) *I*4*/m* phase at 10 GPa. The dashed line at zero indicates Fermi energy.

To examine the nature of the chemical bonds in Ba_4_Au compounds, we calculated the partial density of states for the stable phase at 0, 5, and 10 GPa, as shown in Figure 5. In all three plots, no band gap was observed at the Fermi level. This finding indicates that the electrons at the Fermi level can move freely into the conduction band, showing that the current flow is not limited by a gap. Consequently, all three Ba_4_Au compounds exhibit electrical conductivity and metallic character, thus confirming their classification as metallic compounds. Further analysis of the electronic states near the Fermi level indicates that electrons in the Ba 5*d* orbitals play a significant role in the metallic characteristics of Ba_4_Au. Moreover, we observed a substantial overlap between Ba 5*d* and Au 6*p* orbitals, indicating that Ba–Au bonding mainly originates from the hybridization of Ba 5*d* and Au 6*p* orbitals. Compared with the ambient-pressure *I*4*/mmm* phase (Figure 5a), the *Cmmm* phase at 5 GPa and the *I*4*/m* phase at 10 GPa exhibit a substantial increase in the Au 5*d* density of states near the Fermi level. This behavior indicates that increasing pressure strengthens the covalent interaction between the Ba 5*d* and Au 5*d* orbitals, as evidenced by the enhanced hybridization of these orbitals. Consequently, as external pressure increases, the degree of hybridization among atomic orbitals increases, which enhances the stability of the Ba_4_Au compound under high-pressure conditions.

**Figure 6:**
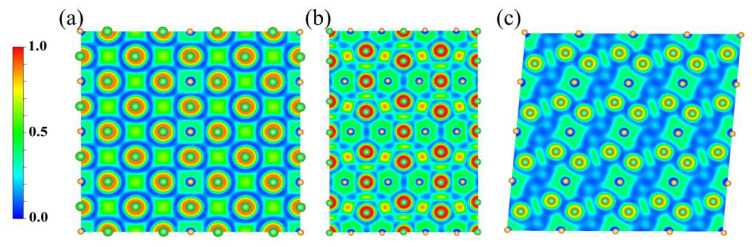
Two-dimensional electron localization function (ELF) diagram of stable structures of Ba_4_Au at varying pressures: (**a**) *I*4*/mmm*-Ba_4_Au at 0 GPa on the (010) plane, (**b**) *Cmmm*-Ba_4_Au at 5 GPa on the (001) plane, and (**c**) *I*4*/m*-Ba_4_Au at 10 GPa on the (100) plane.

Moreover, to investigate the chemical bonding in Ba_4_Au compounds under high pressure, we calculated the ELF of Ba_4_Au compounds at various pressures. The ELF values range from 0 to 1, where 0 indicates fully delocalized electrons or the typical electron deficiency of ionic bonds, 0.5 corresponds to metallic bonding, and 1 denotes fully localized electrons (indicative of covalent bonds) [41,46,47,48]. In the *I*4*/mmm* phase at 0 GPa (Figure 6a), the ELF values between Ba and Au were low, indicating predominantly ionic bonding with charge transfer. The ELF between Ba atoms was approximately 0.5, which is consistent with metallic bonding. At 5 GPa in the *Cmmm* phase (Figure 6b), the ELF around Ba approached one, reflecting strong electron localization. The ELF value between Ba and Au atoms was approximately 0.4, indicating that the Ba–Au bond remains ionic. Compared with the *I*4*/mmm*-Ba_4_Au structure, the Ba–Au ionic bond in the *Cmmm*-Ba_4_Au structure is relatively weaker, with enhanced electron localization between Ba and Au. This behavior correlates with increased hybridization between the Ba 5*d* and Au 5*d* orbitals under pressure. Moreover, ELF between Ba atoms reaches approximately 0.7, indicating a weak covalent characteristic. At 10 GPa in the *I*4*/m* phase (Figure 6c), the Ba–Au interaction remains ionic, whereas Ba–Ba bonding continues to exhibit a metallic characteristic. In summary, ELF indicates that Ba–Au bonds in all three Ba_4_Au structures are ionic, while Ba–Ba bonding varies slightly. In the *I*4*/mmm*-Ba_4_Au and *I*4*/m*-Ba_4_Au structures, Ba–Ba bonds are metallic bonds, whereas in the *Cmmm*-Ba_4_Au structure, Ba–Ba bonds exhibit weak covalent bonding.

**Figure 7:**
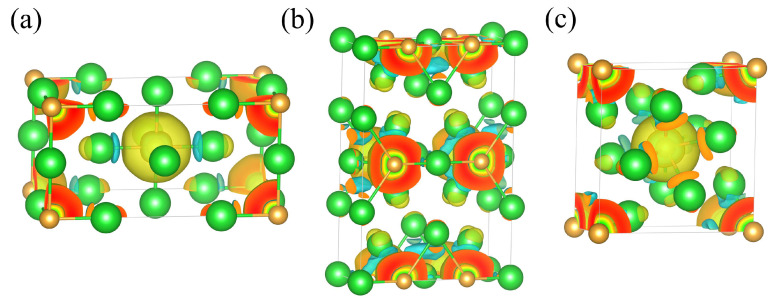
Charge density differences in the (**a**) *I*4*/mmm* (0 GPa), (**b**) *Cmmm* (5 GPa), and (**c**) *I*4*/m* (10 GPa) phases of Ba_4_Au. Blue and yellow regions indicate electron depletion and accumulation, respectively. (Ba in green atoms and Au in gold atoms).

**Figure 8:**
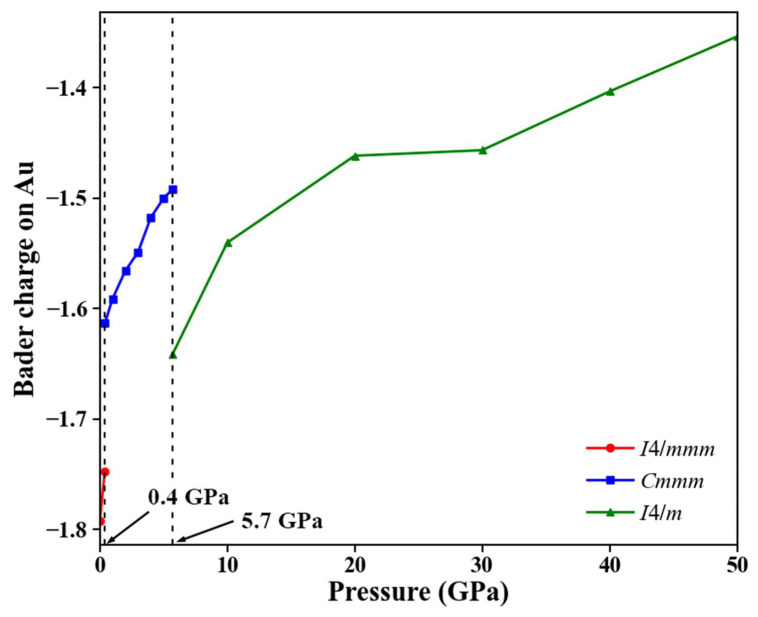
Pressure dependence of Bader charges on Au in each phase within its stable pressure range.

To further investigate the Ba–Au bonding characteristics and charge redistribution, we calculated and analyzed the charge density difference for each phase, as shown in Figure 7. In all three structures, regions of electron depletion and accumulation appeared between Ba and Au atoms, indicating a pronounced charge transfer from Ba to Au. This finding confirms the ionic nature of the Ba–Au bonds in all phases and is consistent with the results from ELF analysis. To quantify the extent of charge transfer between Ba and Au, we conducted Bader charge analysis within the stable pressure ranges of each phase. As shown in Figure 8, in the ambient-pressure *I*4*/mmm* phase, the Bader charge transfer from Ba to Au was approximately 1.79 *e*. At 0.4 GPa, this transfer decreased to approximately 1.75 *e*. With increasing pressure, the Bader charge on Au decreased significantly. Moreover, as pressure increased, the Ba⟶Au charge transfer in each phase progressively diminished, reflecting weakened ionic characteristics and enhanced covalent interactions in the high-pressure structures. This evolution plays a critical role in the stability of Ba_4_Au compounds under compression.

## 4. Conclusions

Herein, we employed the PSO algorithm with first-principles calculations to investigate the pressure-induced structural phase transitions and electronic property evolution in Ba_4_Au. Enthalpy-difference calculations indicated that at ambient pressure, *I*4*/mmm*-Ba_4_Au has the lowest energy. Above 0.4 GPa, the *Cmmm* phase becomes energetically favorable, indicating that the *I*4*/mmm* phase of Ba_4_Au transforms to the *Cmmm* phase at approximately 0.4 Gpa. As pressure increases, above 5.7 GPa, the *I*4*/m* structure becomes energetically more favorable than *Cmmm*-Ba_4_Au, indicating that the *Cmmm* phase transforms to the *I*4*/m* phase at 5.7 GPa. The results of the pressure-induced volume changes revealed that both structural transitions were first-order phase transitions with remarkable volume contractions. The detailed analysis of the Ba_4_Au electronic properties revealed that its three structures exhibited metallic characteristics across various pressures. Furthermore, with increasing pressure, hybridization between the Ba 5*d* and Au 5*d* orbitals progressively strengthened. Bader charge analysis further indicated that as pressure increased, the ionic characteristic between Ba and Au in the high-pressure phases diminished while covalent interactions strengthened. This study not only enriches the high-pressure phase diagram of Ba–Au alloys but also reveals the evolution of the electronic properties of the Ba_4_Au structure under high pressure, indicating its potential applicability in high-pressure electronic devices and pressure-sensitive components with adjustable conductivity.

## Figures and Tables

**Table 1:** Structural parameters of the *I*4*/mmm*, *Cmmm*, and *I*4*/m* phases at varying pressures.

Phase	Pressure (GPa)	Lattice Parameter	Wyckoff Position			
			Atoms	*x*	*y*	*z*
*I*4*/mmm*	0	*a* = *b* = 6.576 Å *c* = 13.165 Å *α* = *β* = *γ* = 90°	Ba1 (4*c*) Ba2 (4*e*) Au (2*a*)	0 0.500 0	0.500 0.500 0	0 0.748 0
*Cmmm*	5	*a* = 11.128 Å *b* = 16.409 Å *c* = 4.312 Å *α* = *β* = *γ* = 90°	Ba1 (2*a*) Ba2 (2*c*) Ba3 (4*i*) Ba4 (8*q*) Au (4*g*)	0 0.500 0 0.800 0.290	1.000 1.000 0.700 0.140 0	0 0.500 0 0.500 1.000
*I*4*/m*	10	*a* = *b* = 9.108 Å *c* = 3.882 Å *α* = *β* = *γ* = 90°	Ba (8*h*) Au (2*a*)	0.082 0	0.728 0	0.500 0

## Data Availability

The original contributions presented in this study are included in the article; further inquiries can be directed to the corresponding author.

## Supplementary Materials

The following supporting information can be downloaded at https://www.mdpi.com/article/10.3390/ma18163728/s1. Table S1: Structural parameters of other Ba_4_Au compounds under various pressure; Figure S1: Phonon spectra of (a) *Pm-*3*m*-Ba_4_Au at 0 GPa, (b) *P*2_1_*/m*-Ba_4_Au at 0 GPa, (c) *P*4*/mmm*-Ba_4_Au at 0 GPa, (d) *C*2-Ba_4_Au at 0 GPa, (e) *Cc*-Ba_4_Au at 0 GPa, (f) *Cmcm*-Ba_4_Au at 10 GPa, and (g) *R-*3*c*-Ba_4_Au at 10 GPa; Table S2: Formation enthalpies of Au*_x_*Ba*_y_* (*x* = 1, *y* = 1, 4; *x* = 2, *y* = 1, 3) compounds under various pressures.

## References

[1] Guenther J., Mallet-Ladeira S., Estevez L., Miqueu K., Amgoune A., Bourissou D. (2014). Activation of Aryl Halides at Gold(I): Practical Synthesis of (P,C) Cyclometalated Gold(III) Complexes. J. Am. Chem. Soc..

[2] Rudolph M., Hashmi A.S.K. (2012). Gold catalysis in total synthesis—An update. Chem. Soc. Rev..

[3] Kodiyath R., Manikandan M., Liu L., Ramesh G.V., Koyasu S., Miyauchi M., Sakuma Y., Tanabe T., Gunji T., Dao T.D. (2014). Visible-light photodecomposition of acetaldehyde by TiO_2_-coated gold nanocages: Plasmon-mediated hot electron transport via defect states. Chem. Commun..

[4] Aragoni M.C., Arca M., Devillanova F.A., Isaia F., Lippolis V., Pintus A. (2011). Gold(III) Complexes of Asymmetrically ArylSubstituted 1,2-Dithiolene Ligands Featuring Potential-Controlled Spectroscopic Properties: An Insight into the Electronic Properties of bis(Pyren-1-yl-ethylene-1,2-dithiolato) Gold(III). Chem.-Asian J..

[5] Zhao W., Yang L., Yin Y., Jin M. (2014). Thermodynamic controlled synthesis of intermetallic Au_3_Cu alloy nanocrystals from Cu microparticles. J. Mater. Chem..

[6] Ishikawa T., Nomura M., Kato K., Suzuki N., Shimizu K., Itoh H. (2013). First-principles study on superconductivity of the gold–indium alloy under high pressure. High Press. Res..

[7] Baranov D.S., Vlaic S., Baptista J., Cofler E., Stolyarov V.S., Roditchev D., Pons S. (2022). Gold Atoms Promote Macroscopic Superconductivity in an Atomic Monolayer of Pb on Si(111). Nano Lett..

[8] Xing Y., Wang H., Li C.K., Zhang X., Liu J., Zhang Y., Luo J., Wang Z., Wang Y., Ling L. (2016). Superconductivity in topologically nontrivial material Au_2_Pb. NPJ Quantum Mater..

[9] Lu J., Zhan M., Yu J., Yu X., Duan Y., Chen S., Xu M., Lu W. (2024). Insight on the Electronic, Elastic and Thermal Properties of Au-Al Intermetallic Compounds Based on First-Principles Calculations. J. Electron. Mater..

[10] Ono S. (2022). Two-Dimensional Ionic Crystals: The Cases of IA-VII Alkali Halides and IA-IB CsAu. J. Phys. Soc. Japan.

[11] Miao M., Brgoch J., Krishnapriyan A., Goldman A., Kurzman J.A., Seshadri R. (2013). On the Stereochemical Inertness of the Auride Lone Pair: Ab Initio Studies of AAu (A = K, Rb, Cs). Inorg. Chem..

[12] Aycibin M., Dogan E.K., Gulebaglan S.E., Secuk M.N., Erdinc B., Akkus H. (2014). Physical properties of RbAu compound. Comput. Condens. Matter.

[13] Lin J., Du X., Yang G. (2019). Pressure-induced new chemistry. Chin. Phys. B.

[14] Lyu T., Yang Q.X., Li Z.M., Zhang C., Liu F., Li J., Hu L., Xu G. (2023). High pressure drives microstructure modification and *zT* enhancement in bismuth telluride-based alloys. ACS Appl. Mater. Interfaces.

[15] Miao M. (2013). Caesium in high oxidation states and as a p-block element. Nat. Chem..

[16] Ma̧czka M., Kryś M., Sobczak S., Vasconcelos D.L.M., Freire P.T.C., Katrusiak A. (2021). Evidence of Pressure-induced phase transitions and negative linear compressibility in formamidinium manganese-hypophosphite hybrid perovskite. J. Phys. Chem. C.

[17] Sun H., Huo M., Hu X., Li J., Liu Z., Han Y., Tang L., Mao Z., Yang P., Wang B. (2023). Signatures of superconductivity near 80 K in a nickelate under high pressure. Nature.

[18] Nelmes R.J., Allan D.R., McMahon M.I., Belmonte S.A. (1999). Self-Hosting Incommensurate Structure of Barium IV. Phys. Rev. Lett..

[19] Li P., Gao G., Wang Y., Ma Y. (2010). Crystal Structures and Exotic Behavior of Magnesium under Pressure. J. Phys. Chem. C.

[20] Rahm M., Cammi R., Ashcroft N.W., Hoffmann R. (2019). Squeezing All Elements in the Periodic Table: Electron Configuration and Electronegativity of the Atoms under Compression. J. Am. Chem. Soc..

[21] Luo D., Wang Y., Yang G., Ma Y. (2018). Barium in High Oxidation States in Pressure-Stabilized Barium Fluorides. J. Phys. Chem. C.

[22] Li F., Zhang X., Fu Y., Wang Y., Bergara A., Yang G. (2021). Ba with Unusual Oxidation States in Ba Chalcogenides under Pressure. J. Phys. Chem. Lett..

[23] Li B., Wang J., Sun S., Liu H. (2022). Crystal Structures and Electronic Properties of BaAu Compound under High Pressure. Materials.

[24] Amsler M., Goedecker S. (2010). Crystal structure prediction using the minima hopping method. J. Chem. Phys..

[25] Laio A., Rodriguez-Fortea A., Gervasio F.L., Ceccarelli M., Parrinello M. (2005). Assessing the accuracy of metadynamics. J. Phys. Chem. B.

[26] Lonie D.C., Zurek E. (2011). XtalOpt: An open-source evolutionary algorithm for crystal structure prediction. Comput. Phys. Commun..

[27] Wang Y., Lv J., Zhu L., Ma Y. (2010). Crystal structure prediction via particle-swarm optimization. Phys. Rev. B.

[28] Pickard C.J., Needs R.J. (2011). Ab initio random structure searching. J. Phys. Condens. Matter.

[29] Peng F., Sun Y., Pickard C.J., Needs R.J., Wu Q., Ma Y. (2017). Hydrogen Clathrate Structures in Rare Earth Hydrides at High Pressures: Possible Route to Room-Temperature Superconductivity. Phys. Rev. Lett..

[30] Liu H., Naumov I.I., Hoffmann R., Ashcroft N.W., Hemley R.J. (2017). Potential High-*T_c_* Superconducting Lanthanum and Yttrium Hydrides at High Pressure. Proc. Natl. Acad. Sci. USA.

[31] Wang H., Wang Y., Lv J., Li Q., Zhang L., Ma Y. (2016). CALYPSO Structure Prediction Method and Its Wide Application. Comput. Mater. Sci..

[32] Li B., Liu H., Liu G., Chen K. (2022). First-principles study on high-pressure phases and compression properties of gold-bearing intermetallic compounds. J. Phys. Condens. Matter.

[33] Wang Y., Lv J., Zhu L., Ma Y. (2012). CALYPSO: A method for crystal structure prediction. Comput. Phys. Commun..

[34] Wei Q., Yang J., Jia X., Luo J., Zhang M., Zhu X. (2025). Crystal structures, mechanical properties, and electronic structure analysis of ternary FeCrAl alloys. Phys. Lett. A.

[35] Yan H., Zhang W., Chen L., Zhang Y., Wang H., Zhang M., Wei Q. (2025). Structural, strength and fracture mechanisms of superconducting transition metal nitrides TM_3_N_5_ (TM= W and Mo). Phys. Chem. Chem. Phys..

[36] Kresse G., Furthmüller J. (1996). Efficient iterative schemes for *ab initio* total-energy calculations using a plane-wave basis set. Phys. Rev. B.

[37] Blöchl P.E. (1994). Projector augmented-wave method. Phys. Rev. B.

[38] Perdew J.P., Zunger A. (1981). Self-interaction correction to density-functional approximations for many-electron systems. Phys. Rev. B.

[39] Perdew J.P., Burke K., Ernzerhof M. (1996). Generalized Gradient Approximation Made Simple. Phys. Rev. Lett..

[40] Savin A., Jepsen O., Flad J., Andersen O.K., Preuss H., Von Schnering H.G. (1992). Electron localization in solid-state structures of the elements: The diamond structure. Angew. Chem. Int. Ed..

[41] Becke A.D., Edgecombe K.E. (1990). A simple measure of electron localization in atomic and molecular systems. J. Chem. Phys..

[42] Togo A., Tanaka I. (2015). First principles phonon calculations in materials science. Scr. Mater..

[43] Chaput L., Togo A., Tanaka I., Hug G. (2011). Phonon-phonon interactions in transition metals. Phys. Rev. B.

[44] Giannozzi P., De Gironcoli S., Pavone P., Baroni S. (1991). *Ab initio* calculation of phonon dispersions in semiconductors. Phys. Rev. B.

[45] Gonze X., Lee C. (1997). Dynamical matrices, Born effective charges, dielectric permittivity tensors, and interatomic force constants from density-functional perturbation theory. Phys. Rev. B.

[46] Savin A., Nesper R., Wengert S., Fässler T.F. (1997). ELF: The Electron Localization Function. Angew. Chem. Int. Ed..

[47] Wang Y.X., Wu H., Xie W.N., Wang X.F., Sun S.W., Gu J.B. (2024). Pressure-induced evolution of structures and phase transition of technetium diboride. J. Appl. Phys..

[48] Xie X., Wei Q., Luo J., Jia X., Zhang M. (2024). Pressure-induced phase transitions of ZrAl_2_ from first-principles calculations. Solid State Commun..

