# Supplementary material for: Pressure-Induced Phase Transitions and Electronic Structure Evolution of Ba4Au
Source: Materials (Basel). 2025 Aug 8;18(16):3728. doi: 10.3390/ma18163728 (PMC12387718; doi:10.3390/ma18163728)
Supplement: Supplementary file 1 [file materials-18-03728-s001.zip › materials-3762782-supplementary.pdf]

Pressure-Induced Phase Transitions and Electronic Structure Evolution of Ba_4Au

Xinyu Wang [1], Qun Wei [1,*], Jing Luo [1], Xiaofei Jia [1], Meiguang Zhang [2,*], Xuanmin Zhu [3], Bing Wei [1,*]

[1] *School of Physics, Xidian University, Xi'an* 710071*, China*
[2] *College of Physics and Optoelectronic Technology, Baoji University of Arts and Sciences, Baoji* 721016*, China*
[3] *School of Information, Guizhou University of Finance and Economics, Guiyang* 550025*, China*

Table S1. Structural parameters of other Ba_4Au compounds under various pressure.

Phase	Pressure (GPa)	Lattice parameter(Å,°)	Wyckoff position				Formation enthalpy (eV/atom)
			Atoms	x	y	z	
Pm-3*m*	0	$a = b = c = 6.5781$	Ba1(1*a*)	0	0	0	-1.5081
		$\alpha = \beta = \gamma = 90$	Ba2(3*c*)	0.500	0	0.500	
			Au(1*b*)	0.500	0.500	0.500	
$P2_1/m$	0	$a = 10.3599$	Ba1(2*e*)	0.989	0.250	0.814	-1.4474
		$b = 4.4501$	Ba2(2*e*)	0.811	0.250	0.443	
		$c = 11.4936$	Ba3(2*e*)	0.677	0.250	0.041	
		$\alpha = \gamma = 90$	Ba4(2*e*)	0.427	0.250	0.310	
		$\beta = 96.07$	Au(2*e*)	0.673	0.750	0.254	
*P*4/*mmm*	0	$a = b = 4.6909$	Ba1(1*c*)	0.500	0.500	0	-1.4268
		$c = 12.8556$	Ba2(1*d*)	0.500	0.500	0.500	
		$\alpha = \beta = \gamma = 90$	Ba3(2*g*)	0	0	0.745	
			Au(1*a*)	0	0	0	
*C*2	0	$a = 15.0397$	Ba1(4*c*)	0.039	0.778	-0.213	-1.4027
		$b = 10.3284$	Ba2(4*c*)	-0.453	0.698	-1.156	
		$c = 10.9341$	Ba3(4*c*)	-0.193	0.466	-0.624	
		$\alpha = \gamma = 90$	Ba4(4*c*)	0.262	0.016	-0.260	
		$\beta = 140.63$	Au(4*c*)	0.169	0.485	-0.105	

		a = 10.1795	Ba1(4*a*)	0.042	0.009	0.235	
		b = 11.9402	Ba2(4*a*)	0.772	0.226	0.965	
Cc	0	c = 8.6346	Ba3(4*a*)	0.723	0.808	0.964	-1.3742
		$\alpha = \gamma = 90$	Ba4(4*a*)	0.449	0.040	0.099	
		$\beta = 92$	Au(4*a*)	0.487	0.786	0.259	
		a = 5.4535	Ba1(4*a*)	0	0	0.500	
		b = 12.1027	Ba2(4*c*)	0.500	0.926	1.250	
Cmcm	10	c = 9.7934	Ba3(8*f*)	0.500	0.198	1.423	-1.7177
		$\alpha = \beta = \gamma = 90$	Au(4*c*)	0	0.165	1.250	
		$a = b$ = 9.7502	Ba1(18*e*)	-0.699	-0.032	0.583	
R-3*c*	10	c = 12.0707	Ba2(6*a*)	-0.667	-0.333	0.917	-1.6882
		$\alpha = \beta = 90$	Au(6*b*)	-0.667	-0.333	0.16	
		$\gamma = 120$					

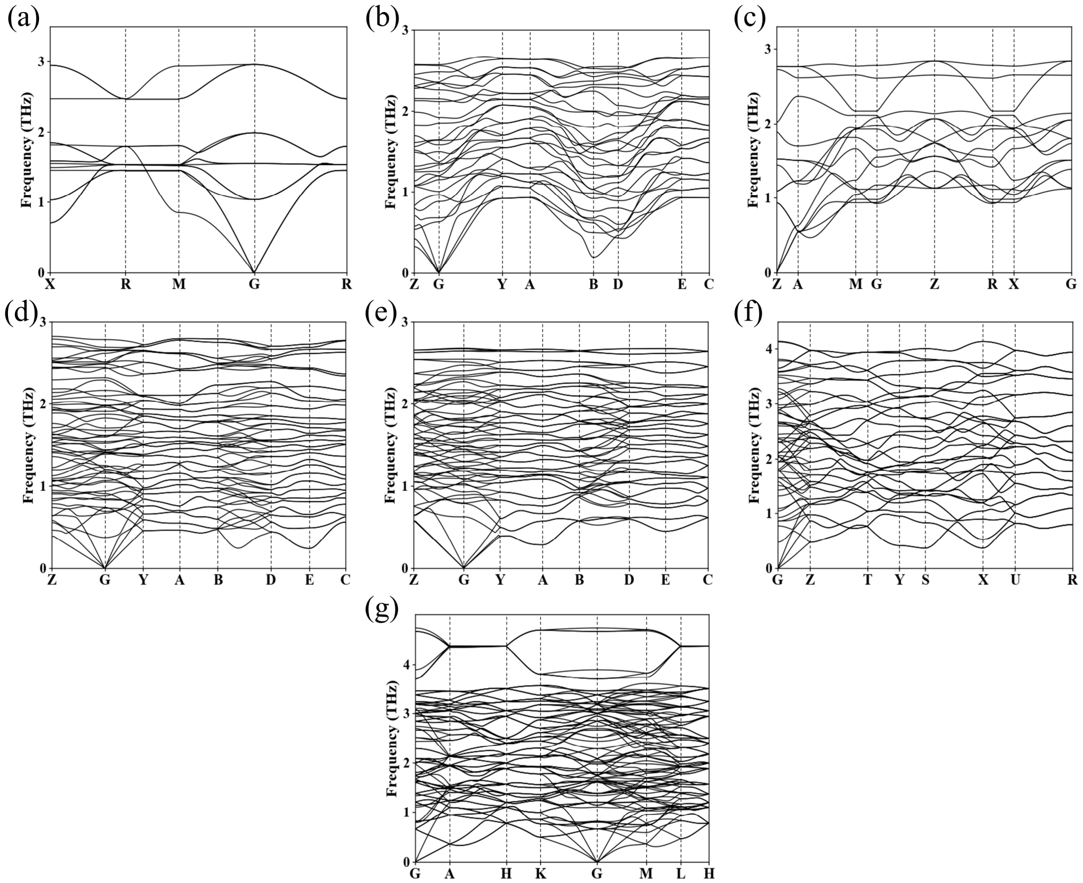

Figure S1. Phonon spectra of (a) *Pm*-3*m*-Ba_4Au at 0 GPa, (b) *P*2_1/*m*-Ba_4Au at 0 GPa, (c) *P*4/*mmm*-Ba_4Au at 0 GPa, (d) *C*2-Ba_4Au at 0 GPa, (e) *Cc*-Ba_4Au at 0 GPa, (f) *Cmcm*-Ba_4Au at 10 GPa, (g) *R*-3*c*-Ba_4Au at 10 GPa.

Table S2. Formation enthalpies of Au_xBa_y (x = 1, y = 1, 4; x = 2, y = 1, 3) compounds under various pressure.

Compound	Pressure (GPa)	Space group	Formation enthalpy (eV/atom)
Au_2Ba	0	*P6/mmm*	-0.7380
Au_2Ba	50	*Fd*-3*m*	-0.4976
AuBa	0	*Pnma*	-0.7204
AuBa	50	*Fd*-3*m*	-0.6118
Au_2Ba_3	0	*R*-3	-0.6030
Au_2Ba_3	50	*Cmcm*	-0.4675
Ba_4Au	0	*I*4/*mmm*	-0.3096
Ba_4Au	50	*I*4/*m*	-0.2488
